# Characterization of the Left Ventricular Myocardium in Systemic Sclerosis

**DOI:** 10.3390/jcm14165627

**Published:** 2025-08-08

**Authors:** Briella K. Egberts, Rajiv Ananthakrishna, Ranjit Shah, Antony Chun Fai So, Jennifer Walker, Sivabaskari Pasupathy, Susanna Proudman, Joseph B. Selvanayagam

**Affiliations:** 1 College of Medicine and Public Health, Flinders University, Bedford Park 5042, Australia; briellaegberts@hotmail.com (B.K.E.); rajiva.ms@gmail.com (R.A.); research@rjshah.com (R.S.); antony.so@sa.gov.au (A.C.F.S.); tharshy.pasupathy@flinders.edu.au (S.P.); 2 Department of Heart Health, South Australian Health & Medical Research Institute, Adelaide 5000, Australia; 3 Department of Cardiovascular Medicine, Flinders Medical Centre, Bedford Park 5042, Australia; 4 Ballarat Base Hospital, Grampians Health, Ballarat 3350, Australia; 5 Wimmera Base Hospital, Horsham 3400, Australia; 6 Discipline of Medicine, University of Adelaide, Adelaide 5000, Australia; jenny.walker@sa.gov.au (J.W.); susanna.proudman@sa.gov.au (S.P.); 7 Rheumatology Unit, Royal Adelaide Hospital, Adelaide 5000, Australia; 8 Faculty of Health Sciences, University of Adelaide, Adelaide 5000, Australia

**Keywords:** systemic sclerosis, coronary microvascular dysfunction, left ventricle, ischemia, SSc-primary heart involvement, oxygen sensitive cardiac magnetic resonance imaging, cardiac magnetic resonance imaging, transthoracic echocardiography

## Abstract

**Background/Objectives:** Cardiac involvement in systemic sclerosis (SSc) ranges from subclinical to severe. While pulmonary artery hypertension (PAH) is well-documented, the mechanism of left ventricular (LV) ischemia remains unclear. Oxygen-sensitive cardiovascular magnetic resonance (OS-CMR) imaging offers a novel approach to assessing myocardial oxygenation and ischemia. This study evaluated the changes in myocardial deoxygenation in response to stress using LV OS-CMR in SSc patients without known cardiac disease. **Methods:** We prospectively recruited SSc patients without prior cardiac disease or risk factors, and age- and sex-matched healthy volunteers (HVs). All participants underwent transthoracic echocardiography (TTE) and 3T CMR, including native T1 mapping, rest/stress OS-CMR, stress perfusion, and late gadolinium enhancement (LGE). The primary outcome was a change in the LV OS-CMR signal intensity (SI) after adenosine stress. **Results:** Thirty-three participants (23 SSc, 10 HV) were enrolled. SSc patients had significantly lower global LV OS-CMR SI compared to HV (13.4 ± 6.5 vs. 19.5 ± 3.6, *p* = 0.011). OS-CMR SI change ≤ 10% was observed in at least one segment in 20 (87%) SSc patients and globally in 12 (52%). LGE was present in 5 (22%) patients, and 18 (78%) had ≥1 abnormal T1 mapping segment. LV global longitudinal strain (GLS) was reduced in SSc patients compared to the HVs (−19.04 ± 3.86 vs. −21.92 ± 3.72, *p* = 0.045). All HVs had normal CMR and TTE findings. **Conclusions:** SSc patients without known cardiovascular disease or PAH demonstrated subclinical LV ischemia with an impaired myocardial oxygenation response to stress. They further demonstrated LV myocardial deformation abnormalities and LV diffuse fibrosis when compared to an age-matched control group. Our findings support the presence of early coronary microvascular dysfunction and LV myocardial fibrosis in this population, which may explain the adverse cardiovascular risk seen in this population, independent of the presence of PAH.

## 1. Introduction

Systemic sclerosis (SSc) is a connective tissue disorder distinguished by widespread vascular abnormalities and notable fibrosis of the skin, and major organ abnormalities [1]. SSc-primary heart involvement (SSc-pHI) (excluding ischemic heart disease (IHD) and pulmonary arterial hypertension (PAH)) develops as a direct consequence of the disease and is one of the most common causes of death in SSc [2]. PAH is well described in this population; however, it has been increasingly recognized that left ventricular (LV) myocardial ischemia may underpin some of these manifestations, and that it may be expressed as a primary feature of the disease. In SSc, distinctive microvascular lesions cause significant dysfunction in the microcirculation, leading to ischemia. Interestingly, however, the prevalence of atherosclerosis in the major coronary arteries in SSc patients appears to be similar to that of the general population [1]. This suggests that while SSc disrupts the microvascular network, it does not necessarily increase the incidence of large-vessel atherosclerosis beyond what is typically observed. Therefore, the cause of left ventricular ischemia may not be driven by coronary artery disease (CAD), but rather by coronary microvascular dysfunction (CMD). Furthermore, animal models have demonstrated occlusive microvascular damage and impaired angiogenesis, referred to as capillary rarefaction [3]. This mechanism of microvascular ischemia is believed to predominate in SSc-associated cardiopulmonary complications [3,4]. A variety of imaging techniques, including transthoracic echocardiography (TTE) and cardiovascular magnetic resonance (CMR), have been used to investigate the prevalence of these cardiac manifestations. Emerging techniques show promise in enhancing this understanding and in reducing mortality and morbidity from SSc-related cardiomyopathies. Oxygen sensitive cardiac magnetic resonance imaging (OS-CMR) is a technique that has been used to directly visualize in vivo myocardial deoxygenation and subsequent ischemia. This technique relies on the effect of oxygenation on the paramagnetic properties of hemoglobin. Deoxygenated hemoglobin serves as an intrinsic contrast agent, affecting the signal intensity in OS-CMR images in a T2/T2*-dependent manner [5]. Changes in signal intensity (SI) in OS-CMR between rest and stress images reflect an inverse relationship with the concentration of deoxygenated hemoglobin. As the concentration of deoxygenated hemoglobin increases, the OS-CMR signal intensity correspondingly decreases. Recently, LV OS-CMR has played a crucial role in advancing our understanding of myocardial ischemia and various LV myocardial [6,7,8] and coronary artery diseases [9,10]. However, its efficacy in assessing LV ischemia in patients with SSc has not been studied. Hence, this is the first study to assess deoxygenation changes in response to stress using OS-CMR in SSc patients with no known cardiac disease, in order to gain a deeper understanding of the mechanisms underlying primary cardiac involvement in SSc.

## 2. Materials and Methods

### 2.1. Study Population

This prospective cross-sectional study recruited male and female participants aged 18 years and older, categorized into two groups: participants with SSc, and age- and gender-matched healthy volunteers (HV). Group 1 consisted of SSc participants who were enrolled in the Australian Scleroderma Cohort Study (ASCS) (Central Adelaide Local Health Network HREC69912) and were attending rheumatology clinics at two South Australian public hospitals between 2021 and 2024. The ASCS enrolls participants who meet the 2013 ACR/EULAR criteria for SSc, with comprehensive data for multiple organ systems collected annually [11]. The HV participants comprised health-care workers who were also prospectively enrolled between 2021 and 2024.

The exclusion criteria for the SSc participants included a clinical or RHC diagnosis of PAH, abnormal transthoracic echocardiogram (TTE), interstitial lung disease (ILD), significant coronary artery disease (>50% stenosis), a history of cardiac disease (e.g., myocardial infarction, angina, cardiomyopathy/heart failure, pulmonary thromboembolism, and significant valvular heart disease), and a clinical diagnosis of obstructive pulmonary disease (no airway obstruction with a pre-bronchodilator FEV1/FVC < 0.7 on recent pulmonary function testing). Group 2 comprised HVs without significant health concerns and with normal CMR and TTE results. The exclusion criteria specific to CMR for all groups included typical CMR contraindications (severe claustrophobia, metal implants including shards of metal in the eye, inability to lie flat for an hour), adenosine contraindications (second- or third-degree atrioventricular block on ECG on the day of the scan), and an estimated glomerular filtration rate (eGFR) of <45 mL/min/1.73 m^2^. All patients underwent a comprehensive transthoracic echocardiogram within three months after their CMR scan. This study was approved by the Southern Adelaide Clinical Human Research Ethics Committee (OFR 269.20) and all participants provided written informed consent.

### 2.2. CMR Protocol

Participants underwent scanning using a 3 Tesla clinical MR scanner (Siemens, Munich, Germany, 3T MAGNETOM VIDA), equipped with an 18-channel torso phased array coil and a spinal coil positioned posteriorly. Subjects refrained from consuming caffeine for 24 h prior to the scan. Using retrospective ECG gating, a breath hold cine balanced steady-state precession sequence (slice thickness 7 mm, TR: 38.6 ms, TE: 1.42 ms, image acquisition matrix 179 × 272, field of view 370 mm, and a flip angle of 80°) was used to acquire three long axis views; a vertical long axis VLA, a horizontal long axis HLA and left ventricular outflow LVOT. Subsequently, these images were used to plan a stack of contiguous short-axis images to cover the entire left and right ventricles. SSFP cine: 7 mm slice, 3 mm gap, TR: 44.2 ms, TE: 1.41 ms, FOV 370 mm, image acquisition matrix 197 × 256, and a flip angle of 80°. A single mid-ventricular short axis slice position was copied for BOLD/OS imaging using a prospective ECG-gated cine OS sequence (slice thickness, 10 mm, TR38.52 ms, TE: 1.44 ms, FOV 400 mm, image acquisition matrix 256 × 243, flip angle 35 degrees). Manual volume shimming was performed immediately prior to OS imaging and frequency adjustments were used as necessary. Each OS-CMR image was obtained during a single breath-hold spanning six heartbeats. Four resting OS-CMR images were acquired before the initiation of adenosine infusion. Acquisition of the OS-CMR stress images began at two minutes following the start of adenosine infusion. Each acquisition lasted on average 8–10 s (dependent on heart rate); we collected four to six OS-CMR acquisitions during the stress period with an average interval of 30 s between acquisitions. Thus, the typical duration of stress OS-CMR image acquisition was approximately three minutes, with a total adenosine infusion duration of about five minutes. Heart rate and blood pressure under stress were recorded every minute during adenosine infusion. Participants were continuously monitored via ECG, sphygmomanometry, and pulse oximetry throughout the study.

### 2.3. CMR Analysis

LV and RV volumes, function, and mass were assessed using specialized software (CVI42 version 5.11.2, Circle Cardiovascular Imaging, Calgary, AB, Canada) with the acquired Short Axis Cine Stack. OS-CMR image analysis was conducted using CVI42. The evaluation of the LV involved the manual tracing of epicardial and endocardial borders (with a 20% offset), which were then subdivided into 6 equiangular segments based on the standard American Heart Association segmentation of the mid-ventricular slice [10]. A blood pool contour was also measured in the LV, making sure no papillary muscles were included. The mean resting myocardial signal intensity (SI) within each segment was determined by averaging the signal measurements from all acquired rest OS-CMR images. Similarly, the mean stress SI was computed by averaging the signal measurements from all stress OS-CMR images obtained during adenosine infusion. Since it was a cardiac-gated sequence, adjustments were made to the mean signal intensity to account for variations in heart rate and their impact on T1 relaxation. The following equation was used for correction for the measured signal intensity for heart rate:

S = S_0_/[1 − βe ^(−TR/T1)^]

where S is the corrected SI and S_0_ is the measured SI. TR is the image-dependent time between the acquisition of sections of k-space, governed by the heart rate. An empirical value of T1 = 1220 ms and β = 0.59 is given. The relative SI change was calculated as follows:

ΔSI (%) = (SI stress − SI rest)/SI rest × 100.

The LV OS-CMR assessment was also evaluated globally by averaging the ΔSI (%) from all 6 LV segments. All OS-CMR analyses were performed by experienced (Society of Cardiovascular Magnetic Resonance Level 3 accredited) observers who were blinded to the group type and clinical information of the participants. Inter-reproducibility was assessed by having the OS-CMR images independently evaluated by two experienced (SCMR Level 3) observers.

### 2.4. Statistical Analysis

Baseline and follow-up data were summarized using descriptive statistics and graphical representations. Discrete variables were summarized using frequencies and percentages, while continuous variables were presented with mean, standard deviation or median with interquartile range (IQR) (Q1, Q3), as deemed appropriate. Percentages were calculated based on the number of participants for whom data were available. For comparisons between three groups, one-way ANOVA or Kruskal–Wallis tests were used. For comparisons between two groups, the independent t-test or Mann–Whitney U test was applied for normally and non-normally distributed continuous variables, respectively. Categorical variables were compared using the Chi-square or Fisher’s exact test. A *p*-value of <0.05 was considered statistically significant. Statistical tests were performed using SPSS version 29. Two experienced independent reviewers performed the CMR analysis, yielding an inter-observer correlation coefficient of 0.8 (*p* < 0.0001). Similarly, transthoracic echocardiogram analyses were performed by two experienced independent reviewers. Inter-observer agreement was high, with an intraclass correlation coefficient (ICC) of 0.93 (*p* < 0.0001), and the intra-observer reproducibility was excellent (ICC = 0.95, *p* < 0.0001), demonstrating robust consistency across reviewers.

## 3. Results

### 3.1. Participant Characteristics

**Figure 1:**
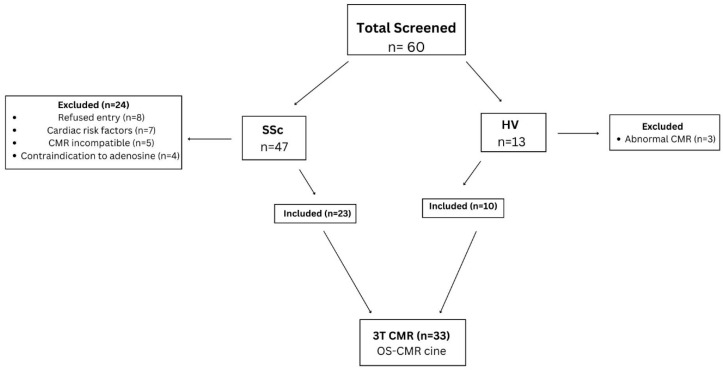
Study Selection. SSc = systemic sclerosis, HV = healthy volunteers.

Of the 60 participants screened, a total of 33 (N = 23 SSc, n = 10 HV) were enrolled (Figure 1) and 27 were excluded. The reasons for exclusion were as follows: patient refused participation (8), pre-existing cardiac disease (7), CMR incompatibility (5) and contraindication to adenosine (4), and HV with abnormal CMR (3). No participants withdrew from the study. Both groups were well matched for age, gender and body surface area (BSA) (Table 1). Among the SSc group (Table 1), 19/23 (83%) of patients had limited cutaneous SSc and the remaining 4/23 (17%) had diffuse SSc, with a mean disease duration of 18 ± 13 years since first non-Raynaud’s symptoms. Further clinical biomarker assessments of SSc patients include median Troponin T of 8 [IQR: 4.5–11.5] and NT-ProBNP 108 [IQR: 69.5–198.5].

### 3.2. CMR Characteristics

Table 2 compares the CMR variables between SSc and HV. There is no statistical difference between the volumetric and functional CMR data between groups; however, there was a significantly lower LV EDVI in the SSc compared to the HV (72.0 ± 14.6 vs. 83.4 ± 12.6, *p* = 0.036) and LV SVI (42.4 ± 8.3 vs. 50.5 ± 9.8, *p* = 0.024). The intraventricular septal thickness was measured at the anteroseptal segment.

Native T1 mapping showed significantly elevated T1 values in SSc compared to HV (1254 ± 38.2 ms vs. 1226 ± 55.5 ms, *p* = 0.049; normal 1235 ms [12]), with 78% of SSc patients having at least one segment with abnormal T1 values. No HV exhibited abnormal T1 values. SSc patients also had significantly higher ECV values than HV (35% ± 11 vs. 28% ± 2.0, *p* = 0.008; normal 22–30% [12]), with 83% showing abnormal ECV values (<30%), whereas no HV exhibited abnormal ECV values. The abnormalities in T1 and ECV suggest increased interstitial fibrosis in SSc patients.

Additionally, all SSc patients displayed replacement fibrosis at the inferior and anterior RV insertion points as evidence of LGE positivity in this area. Five (22%) of the SSc patients exhibited LGE hyperenhancement in the LV: three with midwall LGE in the basal-to-mid inferior septum, one with epicardial-to-midwall LGE in the basal-to-mid lateral wall, and one with subendocardial-to-midwall LGE in the basal inferolateral wall. Notably, all patients with LGE hyperenhancement exhibited the limited form of the disease. No HV showed LGE. Neither group exhibited stress perfusion defects.

### 3.3. Left Ventricular Myocardial Oxygenation Response (OS-CMR)

**Figure 2:**
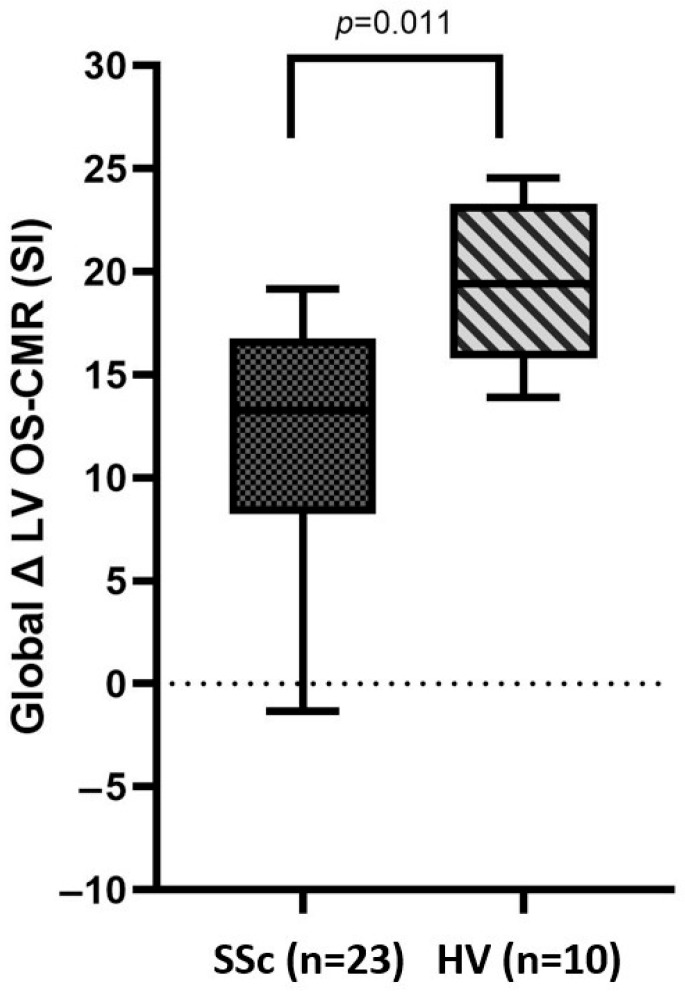
Distribution of global ∆LV OS-CMR SI between SSc and control populations. Here, 18% was the abnormal cut off. *p* < 0.050 indicates statistical significance. SSc = systemic sclerosis; HV = normal volunteer; LV = left ventricle; OS-CMR = oxygen sensitive cardiovascular magnetic resonance; SI = signal intensity.

All participants completed and tolerated the adenosine-induced stress component of the OS-CMR scan. Both groups of patients had a sufficient hemodynamic stress response, as evidenced by a 10% increase from the baseline heart rate and at least one adenosine-related symptom (shortness of breath, chest tightness, flushing and transient headache). All side effects resolved shortly after stopping the adenosine infusion. There was a significantly lower ∆LV OS-CMR SI in the SSc group compared to the HV group (13.4 ± 6.5 vs. 19.5 ± 9.6, *p* = 0.011) (Figure 2). Furthermore, twenty (87%) SSc patients had an LV OS CMR SI change of 10% or less in at least one myocardial segment and 12 (52%) had a global LV OS CMR SI change of at least 10% or less.

After stratifying the SSc group by disease subtype (limited or diffuse), no significant difference was observed in mean ∆LV OS-CMR nor in demographics, clinical biomarkers or medication use. Furthermore, after analyzing the correlation between LV OS-CMR and LV remodeling parameters (LVEF, LVEDV, LVESV, LVSV), no significant associations were found (all *p* values > 0.05).

### 3.4. Echocardiographic Characteristics

All participants underwent an echocardiogram within a mean of 15 days ± 4 of their CMR scan. No significant differences in left ventricular function or volumes were observed between the two groups (*p* > 0.05), indicating a comparable global cardiac performance on conventional assessment. However, the SSc cohort demonstrated a statistically significant reduction in LVEF compared to the HV group (59.16 ± 6.48% vs. 67.75 ± 5.32%, *p* = 0.014), though values remained within the clinically normal range (> 50%) [13]. Similarly, LV-GLS measured by TTE was significantly lower in the SSc compared to the HV group (−19.04 ± 3.86 vs. −21.92 ± 3.72, *p* = 0.045), while still falling within the accepted normal range (>−18%) [13]. Notably, 52% of SSc patients demonstrated abnormal (<−18%) TTE LV-GLS. Among those with abnormal GLS, 42% (5/12) had ≤10%, 33% (4/12) demonstrated LGE, 67% (8/12) showed abnormal T1 mapping and 83% (10/12) had elevated ECV, indicating a high burden of subclinical myocardial abnormalities in this subgroup.

## 4. Discussion

The main finding of this study is that the LV myocardial oxygenation response to adenosine stress is significantly diminished in patients with SSc compared to HV, indicating the presence of myocardial ischemia. Notably, these blunted responses were observed even in SSc patients without prior cardiac disease or confirmed PAH. In addition, SSc patients exhibited subclinical LV myocardial deformation abnormalities and increased interstitial fibrosis. Collectively, these findings provide strong evidence of the presence of global coronary microvascular dysfunction (CMD) in this population. To our knowledge, the application of OS-CMR in patients with SSc and no prior cardiac disease has not been previously explored. Our research advances this field by showing that SSc patients exhibit a reduced myocardial oxygenation response to adenosine stress. These results are indicative of coronary microvascular dysfunction (CMD) in individuals with SSc, even in the absence of established cardiac disease. This offers novel mechanistic insights into the pathophysiology of LV dysfunction in SSc, potentially paving the way for novel treatment approaches for these individuals.

### 4.1. Coronary Microvascular Disease of the LV in SSc Patients

Cardiac involvement induced by SSc is relatively common, occurring in approximately 70% of symptomatic cases and 10–30% of asymptomatic cases. When highly sensitive techniques are used, it is predicted to occur in up to 100% of cases [1]. The pathogenesis of cardiac involvement in SSc is believed to involve myocardial inflammation and persistent coronary microvascular ischemia, which lead to ischemic necrosis, myocardial fibrosis, and reperfusion injury [14]. Pathologic changes within the sub endocardium of small coronary arteries and arterioles predispose the myocardium to ischemia–reperfusion injury, inducing the apoptosis of the cardiomyocytes and eventually replacement fibrosis [15]. Previous CMR studies have explored this phenomenon with the use of a variety of techniques. Stress perfusion techniques highlight that 37.5% of asymptomatic SSc patients exhibit stress perfusion defects [16]. Furthermore, blunted coronary flow reserve (CFR) responses have also supported this, concluding that CMD is likely in SSc patients and is earlier in those affected by the diffuse type and later in those affected by the limited form of the disease [17].

### 4.2. Diffuse Fibrosis

Native CMR T1 parametric mapping is routinely used for the assessment of inflammation/edema and can serve as a proxy for diffuse interstitial fibrosis in the absence of an alternate cause of interstitial expansion (infiltrations/fiber disarray, edema). T1 mapping calculates the total water content in the tissue, and any subtle changes to the intravascular myocardial blood volume (MBV) will prolong the T1 relaxation time, leading to elevated T1 mapping values. In our study, we found a significant difference in native T1 mapping between SSc and normal controls (1254 ms ± 38.2 vs. 1226 ms ± 55.5, *p* = 0.049), with a normal range of <1235 ms [12], with a significant proportion (78%) of the SSc cohort demonstrating values above the normal threshold. Post-contrast T1 mapping has shown benefits in identifying diffuse myocardial fibrosis and aids in calculating the myocardial extracellular volume (ECV) [12]. Our study found significantly higher ECV in SSc patients compared to healthy volunteers, indicating diffuse myocardial interstitial fibrosis. This aligns with Thuny et al. [18], who reported elevated ECV in SSc patients with normal LV function and no LGE, correlating with diastolic dysfunction. TTE LV-GLS can be used as an indirect measure of diffuse fibrosis, and it has been well documented that TTE LV-GLS is independently associated with an increased risk of all-cause mortality and hospitalization in SSc patients [19]. In our study, a significant proportion of the SSc cohort demonstrated TTE LV-GLS values below the normal threshold, indicating a higher prevalence of myocardial deformation in this population despite mean normal values in the group. Furthermore, patients with LV-GLS abnormalities showed a higher prevalence of abnormal ECV and T1 mapping, highlighting the multifactorial nature of myocardial dysfunction in this population.

### 4.3. Study Limitations

Within our study, we only performed a single mid-ventricular cine slice on OS-CMR imaging rather than on multiple slices throughout the entire ventricle. But we trust that in SSc, the myocardium is affected by CMD globally and is not likely to impact the outcome. The LV OS-CMR images were analyzed in mid-diastole, instead of systole. Although studies have shown the ability to acquire and analyze LV OS-CMR images in systole [20], we have chosen to analyze the mid-diastole phase in order to keep it consistent with previously published data in this area, which has shown CMD in the LV [6,21]. Our study also has a relatively small sample size, potentially widening the margin of error. A more extensive study would aid in assessing the effectiveness of these novel techniques in SSc and their correlation with clinical outcomes. Moreover, compared to the SSc group, the sample size in the normal control group was relatively small. Nonetheless, the normal control group was age-matched to the SSc group, reducing any significant bias. Furthermore, our cohort predominantly included limited cutaneous SSc patients, as this is the most common form of the disease, with only a small number of diffuse SSc cases (n = 4), as those with significant cardiac complications were excluded. This likely explains why LGE was observed only in limited SSc and limits the generalizability of our findings to diffuse SSc, where cardiac involvement is often more severe. Larger studies including more diffuse SSc patients are needed to confirm these observations. Although this was a prospective study, we enrolled SSc patients, where some were stable on therapy (ACE, ARB, Beta blockers), which would impact our findings of sub-clinical LV ischemia; however, no correlation was found in patients treated with the medications used and LV OS-CMR. A larger longitudinal follow-up study with clinical outcomes is warranted to assess the clinical implications of these findings and would help to determine the clinical and prognostic utility of these novel CMR techniques in SSc.

### 4.4. Clinical Implications and Future Direction

Our study offers mechanistic insights into the myocardial oxygenation abnormalities in patients with SSc. These findings strongly concur with the presence of CMD, which has significant clinical implications. Patients with CMD may exhibit classic effort-induced angina, along with non-typical symptoms like exertional dyspnea [22]. SSc patients are predisposed to developing PAH; however, this only occurs in 10–12% of patients. Cardiac dysfunction is common in patients with SSc; therefore, there is significant cardiac dysfunction that is not explained by PAH alone. While we have not proven any significant correlation between these novel OS-CMR techniques and conventional prognostic RVEF and hemodynamic parameters, OS-CMR techniques may provide further pathophysiological insights in SSc patients with ongoing unexplainable exertional symptoms. Together, the CMR and TTE findings suggest that subtle myocardial deformation precedes overt cardiac dysfunction in SSc and may have prognostic significance. Given the established links between GLS abnormalities and adverse outcomes, future studies should investigate the integration of biventricular strain analysis into the routine risk stratification and management of SSc patients. Furthermore, characterizing CMD opens up additional possibilities for novel therapies aimed at myocardial ischemia and oxidative stress, such as ranolazine and trimetazidine, which are currently undergoing clinical trials [23]. Although OS-CMR is not intended for routine clinical use, due to its technical complexity (adenosine stress) and limited availability, it remains a powerful research tool for evaluating subclinical ischemia in vivo. In our study, the finding of impaired myocardial oxygenation on OS-CMR was paralleled by abnormalities in the TTE-derived strain, suggesting that more accessible modalities may serve as surrogate markers of CMD. Ultimately, the goal is not to implement OS-CMR as a screening tool, but to use it to inform the development of practical, clinically feasible strategies for early identification and risk stratification in SSc. Future research should focus on translating these mechanistic insights into accessible diagnostic pathways that enable timely intervention in high-risk patients. In this context, OS-CMR serves not as a diagnostic endpoint, but as a foundation to inform and refine the clinical tools of the future.

## 5. Conclusions

Our results indicate that SSc patients demonstrate subclinical left ventricular ischemia compared to healthy volunteers, in the absence of significant LV hypertrophy. Specifically, SSc patients demonstrated a blunted global OS-CMR response to vasodilator stress, consistent with impaired coronary microvascular function. This was accompanied by subtle abnormalities in myocardial deformation and increased interstitial fibrosis, further supporting the presence of diffuse coronary microvascular dysfunction in this population. These observations highlight a potential early cardiac phenotype in SSc, warranting longitudinal follow-up to evaluate clinical outcomes and inform future therapeutic strategies.

## Figures and Tables

**Table 1:** Patient demographics and baseline clinical data.

n (%)	SSc (n = 23)	HV (n = 10)
Age (years, mean ± SD)	60.6 ± 10.2	59.5 ± 6.3
Gender (female (%)	96%	90%
BSA (Mosteller, mean ± SD)	1.75 ± 0.2	1.95 ± 0.3
SSc Specific demographics		N/A
- Disease subtype	Limited = 19 (83%) Diffuse = 4 (17%)	
Disease Length (years, mean ± SD) ^1^	18 ± 13	
- Raynaud’s Phenomenon	23 (100%)	
- ANA antibody + ve	13 (56%)	
- ACA antibody + ve	4 (17%)	
- RNA polymerase antibody + ve	6 (26%)	
- Scl 70 antibody + ve	4 (17%)	
- ASIG Algorithm + ve	3 (13%)	
- Medication		
ACEi/ARB	3 (13%)	N/A
- Beta blocker	7 (30%)	
- Calcium channel blocker	7 (30%)	
- Endothelin receptors blockers	0	
- PDE5 inhibitors	6 (26%)	
- DMARDS (total)	13 (57%)	
- Methotrexate	4 (17%)	
Leflucinide	0	
Sulfasalazine	0	
Hydroxychloroquine	4 (17%)	
Rituximab	2 (9%)	
Infliximab	2 (9%)	
Tocilizumab	1 (4%)	

^1^ from first non-Raynaud’s symptoms. Mann–Whitney U test was used for statistical comparison. *p* < 0.050 indicates statistical significance. SSc = systemic sclerosis, HV = healthy volunteer, BSA = Body Surface Area, ANA = Anti-nuclear antibody, ACA = Anti-centromere antibody, ASIG = Australian Scleroderma Interest Group, ACEi = angiotensin-converting enzyme inhibitors, ARB = angiotensin II receptor blockers, PDE5 = phosphodiesterase-5, DMARDS = Disease Modifying anti-rheumatic drugs.

**Table 2:** CMR volumetric and function data.

Parameter	SSc (n = 23)	HV (n = 10)	*p*-Value
LVEF (%)	60.7 ± 7.0	61.2 ± 4.7	0.501
LV EDVI (ml/m^2^)	72.0 ± 14.6	83.4 ± 12.6	0.036 *
LV ESVI (ml/m^2^)	28.7 ± 9.3	34.7 ± 8.8	0.176
LV SVI (ml/m^2^)	42.4 ± 8.3	50.5 ± 9.8	0.024 *
LV mass index (mg/m^2^)	44.2 ± 6.7	46.1 ± 9.2	0.160
LA max volume index (ml/m^2^)	19.5 ± 4.3	22.2 ± 2.7	0.241
CMR Max IVS (mm)	9.0 ± 1.7	8.8 ± 2.1	0.52
Native T1 Mapping (ms)	1254 ± 38.2	1226 ± 55.5	*p* = 0.049 *
Extra Cellular volume (ECV)	0.35 ± 0.11	0.28 ± 0.02	*p* = 0.008

Mann–Whitney U test was used for statistical comparison. * *p* < 0.050 indicates statistical significance. SSc = systemic sclerosis; HV = normal volunteer CMR = Cardiovascular magnetic resonance, LV = Left ventricle, EF = Ejection Fraction, EDVI = end diastolic volume index, ESVI = end systolic volume index, SVI = stroke volume index, LA = Left Atrium, IVS = intraventricular septum.

## Data Availability

The original contributions presented in this study are included in the article. Further inquiries can be directed to the corresponding author.
